# Supplementary material for: Carboxymethylcytosine is a natural base modification and a handle for bacteriophage DNA hypermodification
Source: Nat Commun. 2025 Dec 7;17:281. doi: 10.1038/s41467-025-66999-9 (PMC12783734; doi:10.1038/s41467-025-66999-9)
Supplement: Supplementary file 1 — Supplementary Information [file 41467_2025_66999_MOESM1_ESM.pdf]

Supplementary Tables

Supplementary Table 1 Pairwise sequence identity and similarity between *Synechococcus* phage S-B43 and *E. coli* K12 proteins.

**S-B43 (uniprot)**	***E. coli* K12 (uniprot)**	**Identity (%)**	**Similarity (%)**	**Alignment length (aa)**	**Key notes**
*Sp*CmoA (A0A514ABH6)	*Ec*CmoA (P76290)	27.9	45.6	272	*Sp*CmoA functions as an orthologous carboxy-SAM synthase.
*Sp*CmoX (A0A514ABG7)	*Ec*DCM (P0AED9)	17.8	30.9	517	*Sp*CmoX is a carboxymethyltransferase functionally diverged from canonical *Ec*DCM.
*Sp*CmoY (A0A514ABJ0)	*Ec*AsnB (P22106)	12.7	20.2	583	*Sp*CmoY contains only ATP-binding domain and lacks the glutamine-hydrolyzing amidase domain present in *Ec*AsnB

Alignment was performed using EMBOSS Needle[1] with the BLOSUM62 substitution matrix. Alignment length includes gap positions introduced during global alignment.

Supplementary Table 2 Oligonucleotides used in this study.

Oligos	Target	Sequence (5' to 3')	Application
p1F	HT-*SpcmoA*	cttccaatccaatatgacttatgatttctcgtttgc	HMT-*SpcmoA*
p1R	HT-*SpcmoA*	gttatccacttccaattacttgatggcaatgactccg	HMT-*SpcmoA*
p2F	Genomic DNA	cttccaatccaattctcaccgcgacacgctattttc	HT-*EccmoA*
p2R	Genomic DNA	tatccacttccaattatgcagcgtcctctgcttttaatg	HT-*EccmoA*
p3F	HT-*SpcmoX*	ccactttaattggaagtggataacggatccg	HT-*SpcmoX*-*SpcmoY*
p3R	HT-*SpcmoX*	ttaaagtttgcttaagacttctttgtaaatg	HT-*SpcmoX*-*SpcmoY*
p4F	HT-*SpcmoY*	taagcaaactttaacgaaattaatacgactcactataggg	HT-*SpcmoX*-*SpcmoY*
p4R	HT-*SpcmoY*	ttccaattaaagtggcagagtcccctgg	HT-*SpcmoX*-*SpcmoY*
p5F	pET28a (+)-HT	tggcgaatgggacgcgccc	110bp dsDNA
p5R	pET28a (+)-HT	aagcgaaaggagcgggcgctag	110bp dsDNA
p6F	Genomic DNA	tctcttttctgaatttgccacctatcatagacagg	*cmoA*-F500
p6R	Genomic DNA	aacgtaaaaatccgggaaaaagaaagtggcgtattg	*cmoA*-F500
p7F	Genomic DNA	ggatttttacgttatgatcgactttggtaacttttattctctgattgcc	*cmoA*-R504
p7R	Genomic DNA	cagtagtttacgcaccgcttcaaactg	*cmoA*-R504
p8F	pRed_Cas9_*recA*	cgccattgaaaacgcatcgagttttagagctagaaatagcaag	pRed_Cas9_*recA*_Δ*cmoA*
p8R	pRed_Cas9_*recA*	ttcagaaaagagactagacgtctgtttctactggtattggc	pRed_Cas9_*recA*_Δ*cmoA*
p9F	pRed_Cas9_*recA*	gcgtaaactactgctagaagcttggattctcacc	pRed_Cas9_*recA*_Δ*cmoA*
p9R	pRed_Cas9_*recA*	tcgatgcgttttcaatggcggcgctagatctgactccataac	pRed_Cas9_*recA*_Δ*cmoA*
p10F	Genomic DNA	cggtacatgctgtactgacggcgac	Sequencing
p10R	Genomic DNA	cgccagcgcggaagggatgaaatagac	Sequencing
p11F	HT-*SpcmoX*	ggagcgtgtgggaaacatggtccccccactgatgatg	HT-*SpcmoX* (R350N)
p11R	HT-*SpcmoX*	tcccacacgctccgtttgctgagag	HT-*SpcmoX* (R350N)

Supplementary Table 3 Palindromic oligonucleotides designed to form double helixes as candidate substrates for *Sp*CmoX

**Oligos**	**Sequence (5' to 3')**
GCGT	GAATT**GCGT**AAAATTTT**ACGC**AATT
GCGG	GAATT**GCGG**AAAATTTT**CCGC**AATT
GCGC	GAATT**GCGC**AAAATTTT**GCGC**AATT
GGCG	GAATT**GGCG**AAAATTTT**CGCC**AATT
CGCG	GAATT**CGCG**AAAATTTT**CGCG**AATT
AGCG	GAATT**AGCG**AAAATTTT**CGCT**AATT
GC	GAATTT**GC**AAAAATTTTT**GC**AAATT
CG	GAATTT**CG**AAAAATTTTT**CG**AAATT
AC	GAATTT**AC**AAAAATTTTT**GT**AAATT
CC	GAATTT**CC**AAAAATTTTT**GG**AAATT

Supplementary Table 4 Data collection and refinement statistics for the crystal of *Sp*CmoX in complex with Cx-SAM

**Data collection**	
Space group	*P 1 21 1*
**Cell dimension (Å)**	
a, b, c (Å)	40.26, 42.13, 98.00
α, β, γ (°)	90.00, 96.05, 90.00
Resolution (Å)	50.00-1.90 (2.00-1.90)
R_{merge}	0.24
$I/\sigma I$	5.3 (0.8)
Completeness (%)	97.3 (100.0)
Redundancy	6.2 (5.1)
$CC_{1/2}$ (%)	99.0 (42.5)
**Refinement**	
Resolution (Å)	48.73-1.90
No. of reflections	25474
R_{work}/R_{free}	0.218/0.264
**No. of atoms**	
Protein	2583
Ligand/ion	30
Water	165
**B-factors**	
Protein	28.8
Water	31.5
Ligand	25.4
**R.M.S. deviations**	
RMSD length (Å)	0.007
RMSD angle (°)	0.836
**Ramachandran plot (%)**	
Favored	96.0
Allowed	3.7
Outliers	0.3

Supplementary Figures

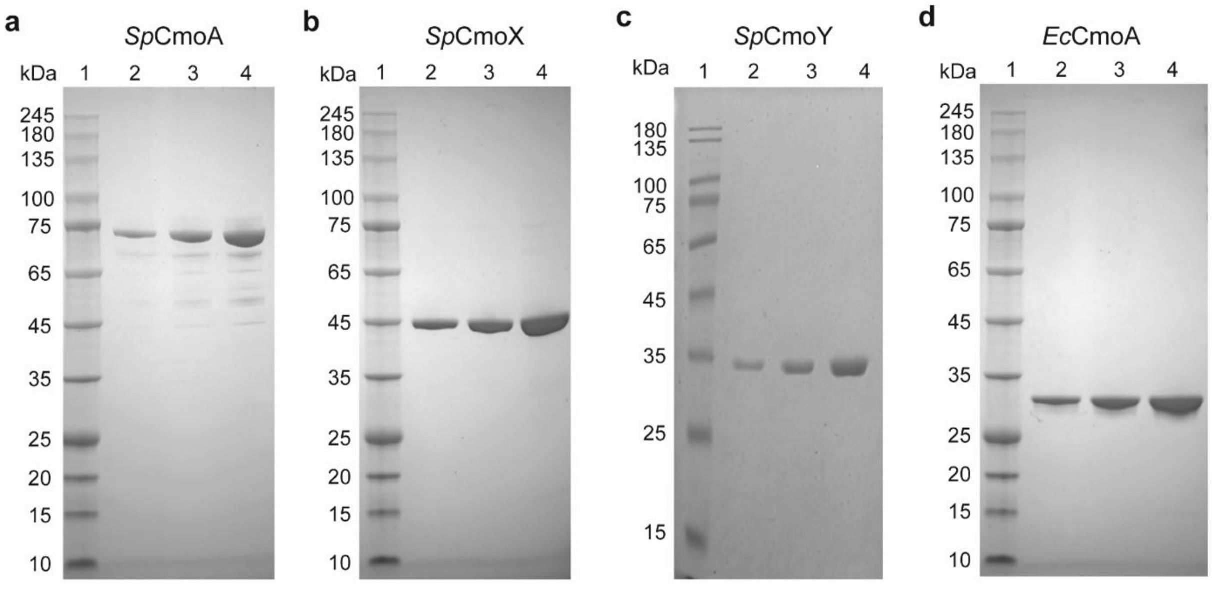

Supplementary Fig. 1 SDS-PAGE analyses of purified proteins. a. MBP-*Sp*CmoA. **b.** *Sp*CmoX. **c.** *Sp*CmoY. **d.** *Ec*CmoA. 1, 2, 4 µg of each purified protein was loaded on lane 2-4 of a gradient gel (8-16%) with the protein molecular weight marker on lane 1.

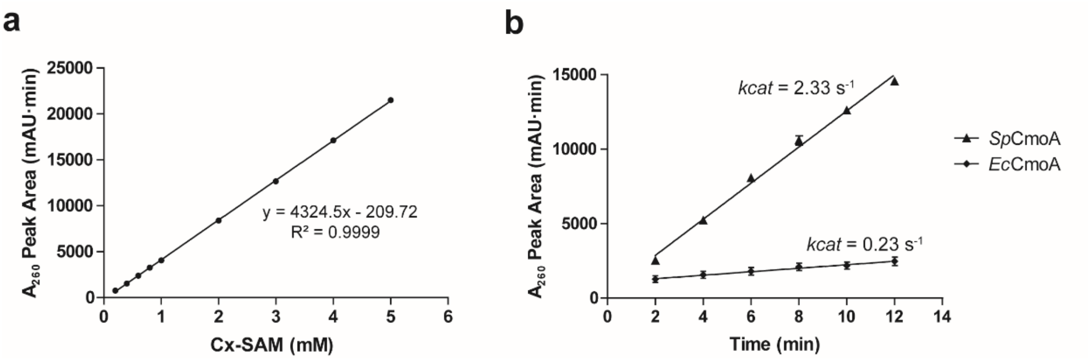

Supplementary Fig. 2 Determination of *kcat* for two homologous CmoA enzymes using UV peak area quantification. **a** A calibration curve of Cx-SAM standard at different concentrations (0-5 mM). **b** Enzymatic activity assay of two homologous enzymes, *Sp*CmoA and *Ec*CmoA, using 10 mM excess substrate and 2 μM enzyme concentration (mean ± s.d., n = 3 independent experiments).

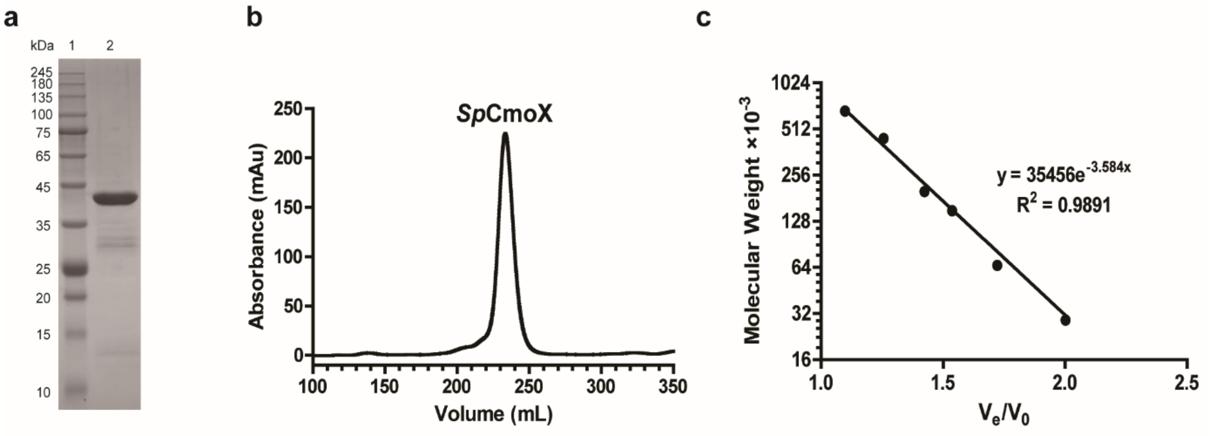

Supplementary Fig. 3 SDS-PAGE and SEC analyses of purified *Sp*CmoX used for protein crystallization. a 12.5% SDS gel with: lane 1, protein molecular weight marker; and lane 2, 6 μg of purified *Sp*CmoX. **b** Elution profile of *Sp*CmoX using Superdex 200 gel filtration chromatography to determine *Sp*CmoX molecular weight, estimated to be 42.9 kDa. **c** Calibration plot based on the molecular weight standards, including bovine thyroglobulin (669 kDa), horse apoferritin (443 kDa), sweet potato β-Amylase (200 kDa), yeast alcohol dehydrogenase (150 kDa), BSA (66 kDa), and bovine carbonic anhydrase (29 kDa) (Sigma MWGF 1000-1KT).

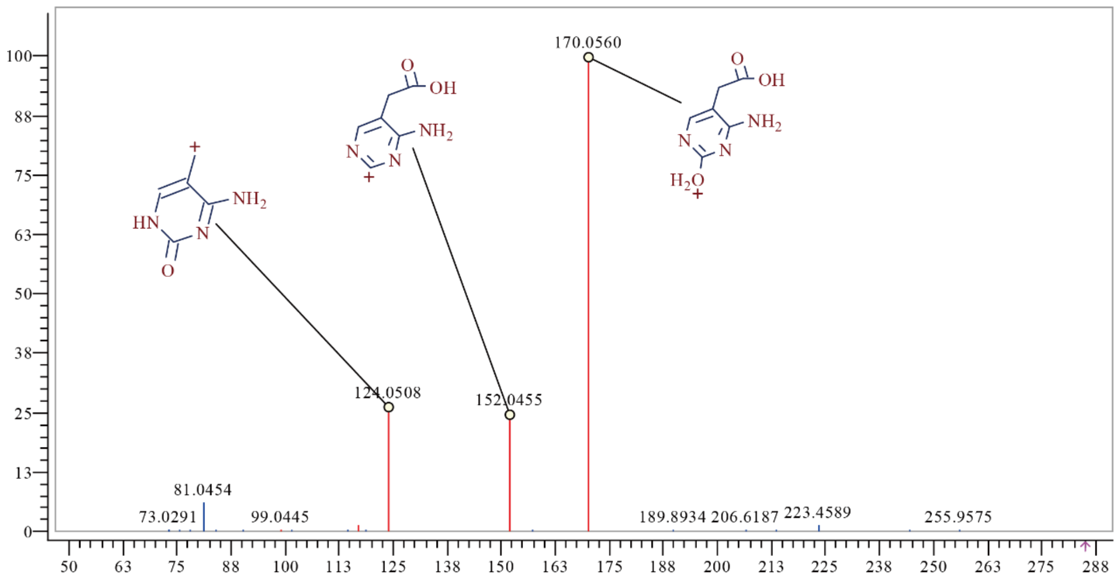

Supplementary Fig. 4 MS/MS analysis of the modified product 5cxmdC.

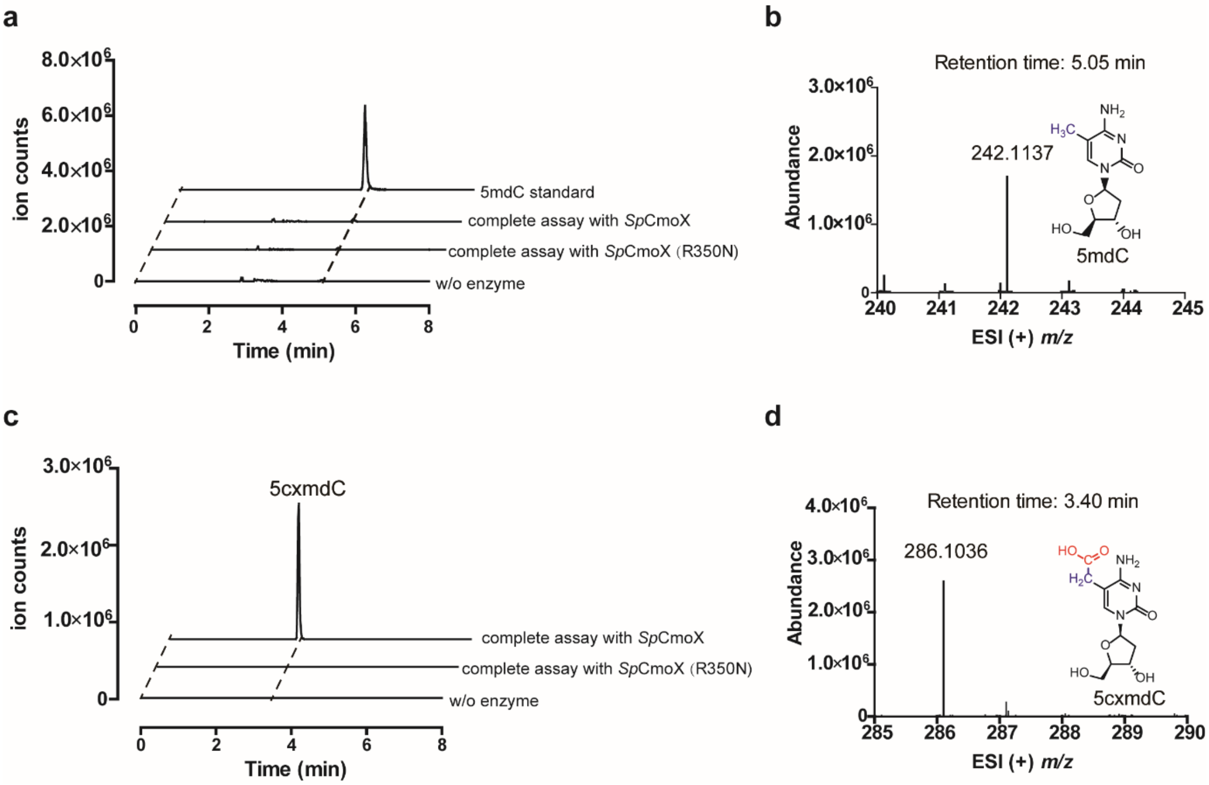

Supplementary Fig. 5 The activity assay of *Sp*CmoX (R350N). a LC-MS extracted ion chromatograms monitoring the formation of 5mdC (*m/z* = 242.1). **b** ESI (+) *m/z* spectrum corresponding to EIC peak for 5mdC. **c** LC-MS extracted ion chromatograms monitoring the formation of 5cxmdC (*m/z* = 286.1). **d** ESI (+) *m/z* spectrum corresponding to EIC peak for 5cxmdC.

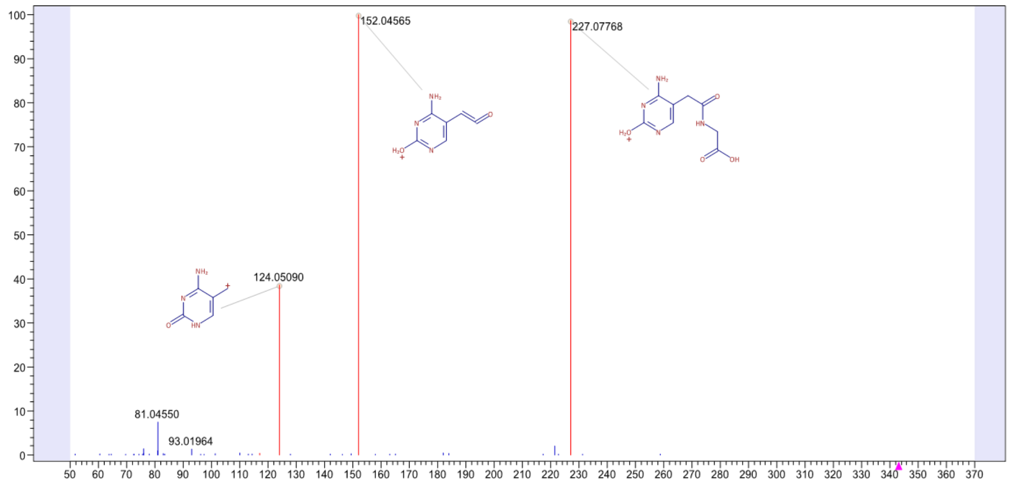

Supplementary Fig. 6 MS/MS analysis of the modified product 5gcxmdC.

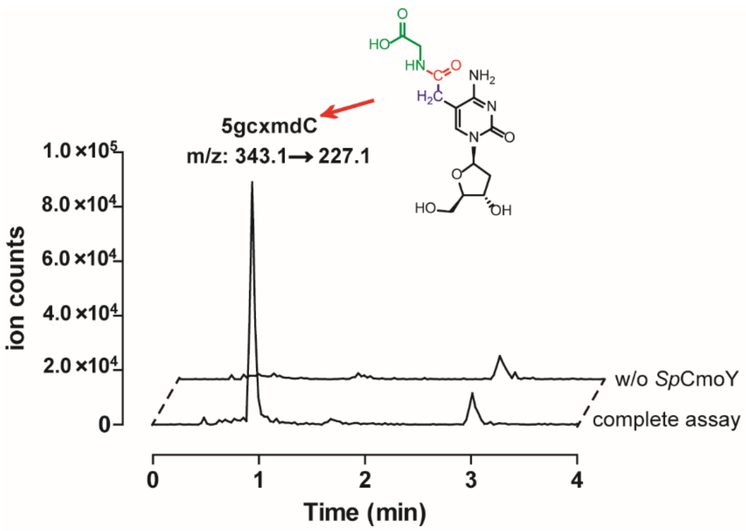

Supplementary Fig. 7 LC-MS/MS analysis of 5gcxmdC formation from 110-bp dsDNA containing 5cxmC. LC-MS/MS extracted ion chromatograms were employed to monitor the formation of 5gcxmdC in an *in vitro* reaction using 110-bp dsDNA containing 5cxmC as the substrate.

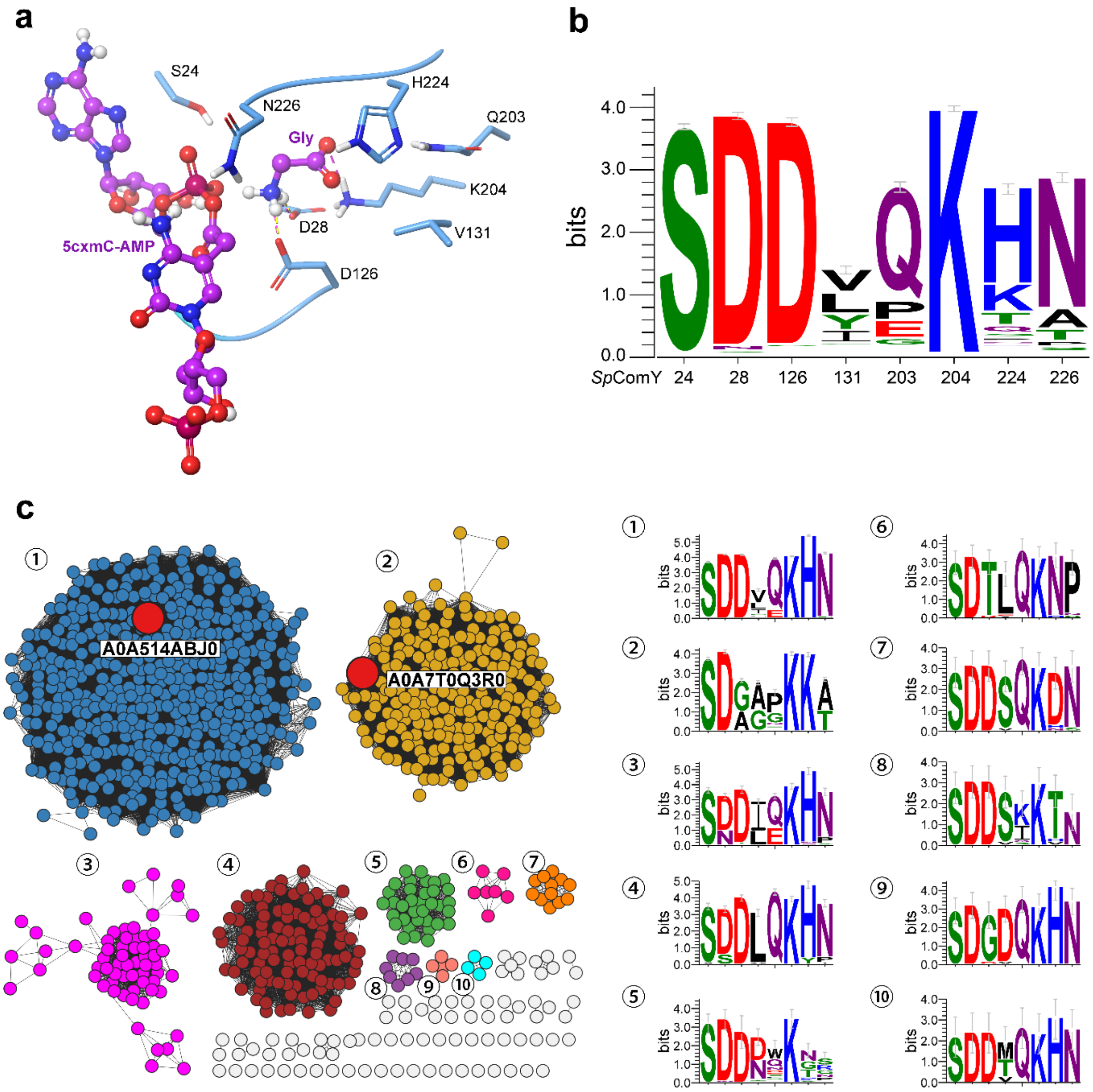

Supplementary Fig. 8 Diversity of the ligand-binding pocket in AMP-forming ligases. a Predicted structure model of *Sp*CmoY bound to glycine and 5cxmC-AMP, showing the glycine-binding pocket. The structure was generated using AlphaFold 3 and used to visualize the predicted ligand binding mode. **b** Weblogo of ligand-binding pocket residues across 2,251 AMP-forming ligases. **c** SSN of the 2,251 AMP-forming ligases (reproduced from Fig. 8f), with cluster-specific Weblogos highlighting differences in ligand-binding residue composition.

Supplementary Tables

Supplementary Table 1 Pairwise sequence identity and similarity between *Synechococcus* phage S-B43 and *E. coli* K12 proteins.

**S-B43 (uniprot)**	***E. coli* K12 (uniprot)**	**Identity (%)**	**Similarity (%)**	**Alignment length (aa)**	**Key notes**
*Sp*CmoA (A0A514ABH6)	*Ec*CmoA (P76290)	27.9	45.6	272	*Sp*CmoA functions as an orthologous carboxy-SAM synthase.
*Sp*CmoX (A0A514ABG7)	*Ec*DCM (P0AED9)	17.8	30.9	517	*Sp*CmoX is a carboxymethyltransferase functionally diverged from canonical *Ec*DCM.
*Sp*CmoY (A0A514ABJ0)	*Ec*AsnB (P22106)	12.7	20.2	583	*Sp*CmoY contains only ATP-binding domain and lacks the glutamine-hydrolyzing amidase domain present in *Ec*AsnB

Alignment was performed using EMBOSS Needle[1] with the BLOSUM62 substitution matrix. Alignment length includes gap positions introduced during global alignment.

Supplementary Table 2 Oligonucleotides used in this study.

Oligos	Target	Sequence (5' to 3')	Application
p1F	HT-*SpcmoA*	cttccaatccaatatgacttatgatttctcgtttgc	HMT-*SpcmoA*
p1R	HT-*SpcmoA*	gttatccacttccaattacttgatggcaatgactccg	HMT-*SpcmoA*
p2F	Genomic DNA	cttccaatccaattctcaccgcgacacgctattttc	HT-*EccmoA*
p2R	Genomic DNA	tatccacttccaattatgcagcgtcctctgcttttaatg	HT-*EccmoA*
p3F	HT-*SpcmoX*	ccactttaattggaagtggataacggatccg	HT-*SpcmoX*-*SpcmoY*
p3R	HT-*SpcmoX*	ttaaagtttgcttaagacttctttgtaaatg	HT-*SpcmoX*-*SpcmoY*
p4F	HT-*SpcmoY*	taagcaaactttaacgaaattaatacgactcactataggg	HT-*SpcmoX*-*SpcmoY*
p4R	HT-*SpcmoY*	ttccaattaaagtggcagagtcccctgg	HT-*SpcmoX*-*SpcmoY*
p5F	pET28a (+)-HT	tggcgaatgggacgcgccc	110bp dsDNA
p5R	pET28a (+)-HT	aagcgaaaggagcgggcgctag	110bp dsDNA
p6F	Genomic DNA	tctcttttctgaatttgccacctatcatagacagg	*cmoA*-F500
p6R	Genomic DNA	aacgtaaaaatccgggaaaaagaaagtggcgtattg	*cmoA*-F500
p7F	Genomic DNA	ggatttttacgttatgatcgactttggtaacttttattctctgattgcc	*cmoA*-R504
p7R	Genomic DNA	cagtagtttacgcaccgcttcaaactg	*cmoA*-R504
p8F	pRed_Cas9_*recA*	cgccattgaaaacgcatcgagttttagagctagaaatagcaag	pRed_Cas9_*recA*_Δ*cmoA*
p8R	pRed_Cas9_*recA*	ttcagaaaagagactagacgtctgtttctactggtattggc	pRed_Cas9_*recA*_Δ*cmoA*
p9F	pRed_Cas9_*recA*	gcgtaaactactgctagaagcttggattctcacc	pRed_Cas9_*recA*_Δ*cmoA*
p9R	pRed_Cas9_*recA*	tcgatgcgttttcaatggcggcgctagatctgactccataac	pRed_Cas9_*recA*_Δ*cmoA*
p10F	Genomic DNA	cggtacatgctgtactgacggcgac	Sequencing
p10R	Genomic DNA	cgccagcgcggaagggatgaaatagac	Sequencing
p11F	HT-*SpcmoX*	ggagcgtgtgggaaacatggtccccccactgatgatg	HT-*SpcmoX* (R350N)
p11R	HT-*SpcmoX*	tcccacacgctccgtttgctgagag	HT-*SpcmoX* (R350N)

Supplementary Table 3 Palindromic oligonucleotides designed to form double helixes as candidate substrates for *Sp*CmoX

**Oligos**	**Sequence (5' to 3')**
GCGT	GAATT**GCGT**AAAATTTT**ACGC**AATT
GCGG	GAATT**GCGG**AAAATTTT**CCGC**AATT
GCGC	GAATT**GCGC**AAAATTTT**GCGC**AATT
GGCG	GAATT**GGCG**AAAATTTT**CGCC**AATT
CGCG	GAATT**CGCG**AAAATTTT**CGCG**AATT
AGCG	GAATT**AGCG**AAAATTTT**CGCT**AATT
GC	GAATTT**GC**AAAAATTTTT**GC**AAATT
CG	GAATTT**CG**AAAAATTTTT**CG**AAATT
AC	GAATTT**AC**AAAAATTTTT**GT**AAATT
CC	GAATTT**CC**AAAAATTTTT**GG**AAATT

Supplementary Table 4 Data collection and refinement statistics for the crystal of *Sp*CmoX in complex with Cx-SAM

**Data collection**	
Space group	*P 1 21 1*
**Cell dimension (Å)**	
a, b, c (Å)	40.26, 42.13, 98.00
α, β, γ (°)	90.00, 96.05, 90.00
Resolution (Å)	50.00-1.90 (2.00-1.90)
R_{merge}	0.24
$I/\sigma I$	5.3 (0.8)
Completeness (%)	97.3 (100.0)
Redundancy	6.2 (5.1)
$CC_{1/2}$ (%)	99.0 (42.5)
**Refinement**	
Resolution (Å)	48.73-1.90
No. of reflections	25474
R_{work}/R_{free}	0.218/0.264
**No. of atoms**	
Protein	2583
Ligand/ion	30
Water	165
**B-factors**	
Protein	28.8
Water	31.5
Ligand	25.4
**R.M.S. deviations**	
RMSD length (Å)	0.007
RMSD angle (°)	0.836
**Ramachandran plot (%)**	
Favored	96.0
Allowed	3.7
Outliers	0.3

Supplementary References

1. Madeira F*, et al.* The EMBL-EBI Job Dispatcher sequence analysis tools framework in 2024. *Nucleic acids research* **52**, W521-W525 (2024).
